# Ectopic Expression of *OLEOSIN 1* and Inactivation of *GBSS1* Have a Synergistic Effect on Oil Accumulation in Plant Leaves

**DOI:** 10.3390/plants10030513

**Published:** 2021-03-09

**Authors:** Zhiyang Zhai, Hui Liu, John Shanklin

**Affiliations:** Biology Department, Brookhaven National Laboratory, BNL 463, 50 Bell Ave., Upton, NY 11953, USA; huiliu1@bnl.gov

**Keywords:** triacylglycerol, fatty acids, oleosin, granule bound starch synthase, metabolic engineering

## Abstract

During the transformation of wild-type (WT) *Arabidopsis thaliana*, a T-DNA containing *OLEOSIN-GFP* (*OLE1-GFP*) was inserted by happenstance within the *GBSS1* gene, resulting in significant reduction in amylose and increase in leaf oil content in the transgenic line (OG). The synergistic effect on oil accumulation of combining *gbss1* with the expression of *OLE1-GFP* was confirmed by transforming an independent *gbss1* mutant (GABI_914G01) with *OLE1-GFP.* The resulting *OLE1-GFP*/*gbss1* transgenic lines showed higher leaf oil content than the individual *OLE1-GFP/*WT or single *gbss1* mutant lines. Further stacking of the lipogenic factors WRINKLED1, Diacylglycerol O-Acyltransferase (DGAT1), and Cys-OLEOSIN1 (an engineered sesame OLEOSIN1) in *OG* significantly elevated its oil content in mature leaves to 2.3% of dry weight, which is 15 times higher than that in WT Arabidopsis. Inducible expression of the same lipogenic factors was shown to be an effective strategy for triacylglycerol (TAG) accumulation without incurring growth, development, and yield penalties.

## 1. Introduction

The oil triacylglycerol (TAG) is an energy-dense molecule that is essential for human nutrition and constitutes feedstocks for the production of biofuels and bioproducts [1]. TAGs consist of three fatty acids esterified to a single molecule of glycerol. Photosynthetically derived sugars provide carbon skeletons, energy, and reductant for fatty acid (FA) and TAG synthesis. In plants, multiple enzymes (including acetyl-CoA carboxylase, fatty acid synthase, acyltransferases, etc.) work in concert to catalyze de novo fatty acid synthesis and TAG assembly [2,3]. TAG mainly accumulates in plant seeds. Vegetative tissues, which constitute a major portion of plant biomass, have very low levels (typically less than 0.1% of dry weight) of TAG. Recent efforts in genetic and metabolic engineering have resulted in the successful accumulation of TAG in the leaves of both dicot and monocot crop plants [4,5]. For example, the Push-Pull-Protect strategy resulted in the accumulation of significant levels of TAG in tobacco leaves [6] by ectopic co-expression of *WRINKLED1* (encoding WRI1, a master transcriptional regulator of glycolysis and fatty acid synthesis [7,8]), *DGAT1* (encoding diacylglycerol acyltransferase 1, which catalyzes the final step of TAG biosynthesis [9]), and *OLEOSIN1* (*OLE1*, encoding a structural oil body protein [10]), in which WRI1 is for pushing, DGAT1 is for pulling, and OLE1 is for protecting oil. Reducing fatty acid (or TAG) degradation and diverting carbon from starch synthesis also favors oil accumulation. In Arabidopsis, it was found that overexpression of *WRI1* alone resulted in a 2.6–2.8-fold increase in TAG accumulation in leaves. Suppression of the small subunit of Adenosine diphosphate (ADP)-glucose pyrophosphorylase (ADG1), which catalyzes the first committed step of starch synthesis, by RNAi in wild type (WT) only slightly increased TAG accumulation. However, the combination of *WRI* overexpression along with *ADG1 RNAi* produced 5.8 times more TAG than WT [11]. *WRI1*, *DGAT1,* and *OLE1* overexpression with simultaneous silencing of *ADG1* and *PXA1*, an ABC transporter-like protein required for fatty acid β-oxidation in peroxisome [12], produced a transgenic sugarcane that generated 95 times more TAG in leaves than WT [13]. Mutation of *SDP1* encoding a TAG lipase also significantly boosts vegetative oil accumulation when *WRI1* and *DGAT1* are co-expressed in Arabidopsis [14]. Another successful strategy for promoting vegetative oil production is to change sugar partitioning. *SUC2* encodes a membrane sucrose-proton symporter that is essential for sucrose phloem loading and long-distance transport [15]. The *suc2 adg1* double mutant was shown to accumulate an 80-fold higher level of soluble sugars (sucrose, glucose, and fructose combined) in leaves compared to WT. Correspondingly, the high sugar content in *suc2 adg1* boosted TAG accumulation to more than 1% of dry weight (DW) [16].

Starch is the major storage carbohydrate in plants. It is accumulated at high levels as granules in storage tissues such as grains, tubers, and roots, and deposited in most vegetative tissues to a lesser extent. The dominant components of starch granules are two types of glucan polymer: amylose (making up ~30%) and amylopectin (which constitutes ~70%). Lipids represent the most important minor component associated with the starch granules [17,18]. The lipids associated with starch granules can be surface lipids or internal lipids. TAG is a major component of surface lipids in maize and wheat starch granules. Other surface lipids including glycolipids and phospholipids derive from the amyloplast membrane [19]. In contrast, internal lipids are mainly composed of monoacyl lipids (free fatty acid and lysophospholipids) in amounts that are proportional to the amylose content [20]. The starch biosynthesis pathway is well established, involving ADP-glucose pyrophosphorylase, starch synthase (SS), and starch branching enzyme (SBE). ADP-glucose, which is synthesized from glucose-1-phosphate and ATP in a reaction catalyzed by ADP-glucose pyrophosphorylase, is the precursor for both amylose and amylopectin. In the remaining steps of starch synthesis, SS catalyzes the synthesis of an α (1→4) linkage. The α (1→6) branches in starch polymers are brought by SBE. Two different types of SS are present in plants: granule-bound SS (GBSS) and soluble SSs. GBSS is responsible for amylose biosynthesis, while soluble SSs mainly synthesize amylopectin [17,18].

In the current research, characterization of a high-leaf-oil *OLE1-GFP* transgenic line (named *OG* hereafter) showed that T-DNA inserted within the *GBSS1* gene disrupted the only granule-bound starch synthase gene in Arabidopsis. Overexpression of *OLE1-GFP* in an independent *gbss1* mutant resulted in significantly higher leaf TAG content than its expression in WT or *gbss1* without expression of further transgenes. Unlike *OLE1-GFP*/WT transgenic plants, which showed growth defects, *OG* plants are visibly indistinguishable from WT, making it a strong candidate line for further engineering of vegetative oil. Stacking of *WRINKLED1, DGAT1,* and *Cys-OLE1* (encoding an engineered sesame OLE1 [21]) in *OG* further increased leaf TAG content to 2.26% of DW, demonstrating that deficiency of *GBSS1* is compatible with commonly employed Push-Pull-Protect strategies [6] for boosting vegetative oil accumulation.

## 2. Results

### 2.1. An Arabidopsis OLE1-GFP Transgenic Line with a T-DNA Insertion in GBSS1 Accumulated Significant Leaf TAG

**Figure 1:**
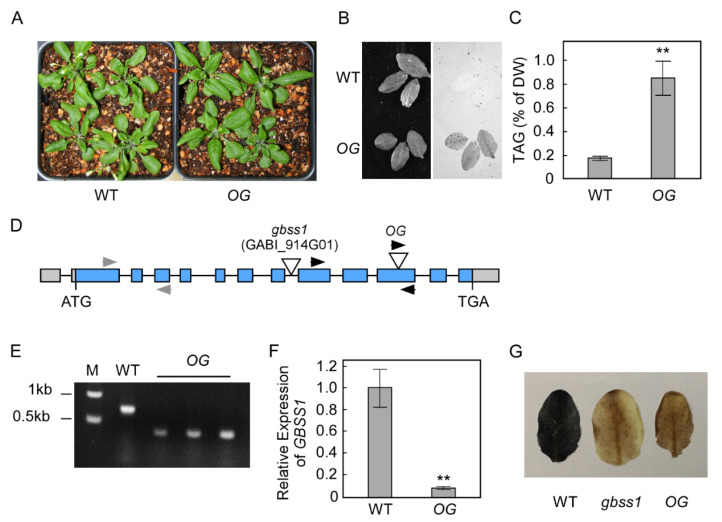
Characterization of an *Arabidopsis OLE1*-*GFP* transgenic line (*OG*). (**A**) Phenotype of 1-month-old wild type (WT) and *OG* plants. (**B**) Bright light (Left) and GFP fluorescence (Right) of WT and *OG* by fluorescence image analyzer (ImageQuant LAS4000). (**C**) Leaf TAG contents of WT plants (WT) and *OG*. Values are means ± SE (*n* = 6) of measurements on mature leaves of 1-month-old soil grown plants. Asterisks denote statistically significant difference from WT (Student’s *t* test, **, *p* < 0.01). (**D**) Schematic illustration of the exon-intron structure of the *GBSS1* gene. Exons are represented by blue boxes. Grey boxes represent the 5′ and 3′ UTRs. Translation start (ATG) and stop (TAG) codons are indicated. Open inverted triangles indicate T-DNA insertion sites for *OG* and *gbss1* mutant respectively. Black arrowheads indicate primers for genotyping *OG*. Grey arrowheads represent primers used in quantitative real time PCR (qRT-PCR) for quantifying gene expression of *GBSS1*. (**E**) Genotyping of *OG* with primers in (**D**). M is DNA marker. (**F**) Analysis of *GBSS1* expression in WT and *OG* plants. The values are means ± SE of measurements taken by qRT-PCR on three individual plants employing primers (gray arrow) shown in (**D**). F-box expression was used as a control for normalization (**, *p* < 0.01). (**G**) Iodine staining of leaves starch granules isolated from 0.5 g (FW) leaves of WT and *OG* plants.

Here we characterize a previously described *Arabidopsis OLE1-GFP* transgenic line called OG [22]. The transgenic line was generated by transforming *Arabidopsis* WT (WT) plant with the binary vector pGKPGWG containing the *OLE1* genomic sequence with an in-frame GFP C-terminal extension under the control of the CaMV35S promoter. While the growth of *OG* is similar to that of WT plants, its mature leaves showed strong GFP fluorescence signal and accumulated over 4 times more TAG than WT plants (Figure 1A–C), Winichayakul et al. had shown little if any TAG increase in WT *Arabidopsis* leaves overexpressing sesame *OLE* [21]. In other work, *Arabidopsis* plants expressing CaMV35S:*OLE1-GFP* did not accumulate significant amounts of fusion proteins in the leaves either [23]. Other WT *OLE1-GFP* transgenics transformed with the same construct as *OG* have shorter siliques and lower GFP fluorescence signal relative to *OG* (Supplementary Figure S1). Transient expression of the same construct was also conducted in *Nicotiana benthamiana* (*N. benthamiana*) leaves. Three independent experiments showed that expression of Ole1-GFP increased the leaf TAG content by 1.4-, 1.6- and 2.3-fold compared with empty vector controls (Supplementary Figure S2). To explain these apparently conflicting results, we hypothesized that the T-DNA insertion site of *OG* might be contributing to the observed high leaf TAG in *OG*. Crosses with WT *OG*×Col0 resulted in F2 seeds with a 3:1 resistant:susceptible Kanamycin phenotype indicating that *OG* contains a single T-DNA locus of insertion. Thermal asymmetric interlaced PCR (TAIL-PCR) was utilized to identify the sequence flanking the OG T-DNA insertion site. At the borders of the T-DNA insert site we identified a sequence encoding the 10th exon of *GBSS1* (Figure 1D). Genotyping further confirmed *GBSS1* was disrupted in *OG* by using PCR primers designed against either side of insertion and the left border of the T-DNA construct (Figure 1E). Quantification of expression of *GBSS1* in leaves of *OG* showed that its expression was reduced by more than 90% compared to WT (Figure 1F). *GBSS1* is the only starch synthase capable of catalyzing the synthesis of amylose in starch granules of the chloroplast. Iodine staining of *OG* leaves was similar to that of an independent *gbss1* mutant (GABI_914G01 [24]), confirming that most if not all of the amylose was depleted and that the function encoded by *GBSS1* in *OG* was indeed compromised by the T-DNA insertion (Figure 1G). In sum, we found *OG* had a unique genetic background resulting from the insertion of the *OLE 1-GFP* overexpression T-DNA in the *GBSS1* gene, thereby creating a GBSS1-deficient background.

### 2.2. Overexpression of OLE1 in a gbss1 Background Resulted in Higher TAG Accumulation Than in WT

**Figure 2:**
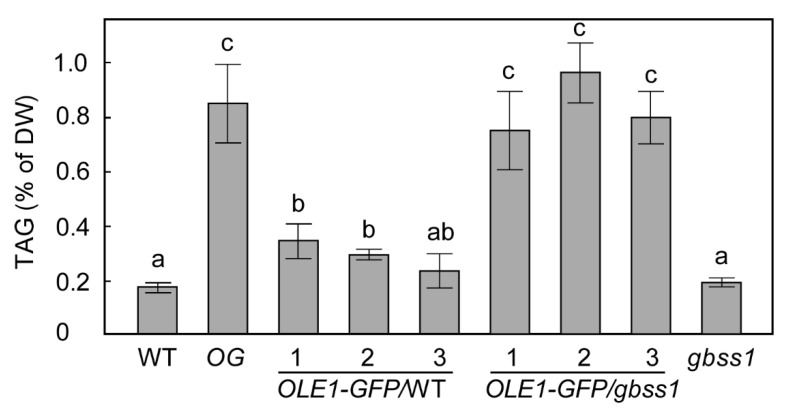
Overexpression of *OLE1-GFP* and *gbss1* mutation have synergistic roles in increasing TAG content in leaves. TAG quantification in leaves of varied genotypes as indicated. Values are means ± SE of measurements on mature leaves of 1-month-old soil grown plants. Levels indicated with different letters above histogram bars are significantly different (Student’s *t* test for all pairs of genotypes, *n* = 6, *p* < 0.05).

Increased accumulation of TAG in *OG* suggested that deficiency of GBSS1 together with ectopic expression of *OLE1* may have synergistic roles in increasing leaf TAG synthesis. To test this hypothesis, an independent *gbss1* mutant (GABI_914G01 [24], Figure 1B) (and WT as a control) were transformed with *OLE1-GFP* driven by the CaMV35S promoter. More than 10 independent transgenic lines for both genetic backgrounds were identified. Three randomly selected transgenic lines for each background were taken to the T3 generation to identify homozygous lines. TAG quantification in mature leaves of 4-week-old plants showed that while the TAG content of *gbss1* was no different from WT, TAG in *OLE-GFP*/*gbss1* was significantly higher than either that of OL*E1-GFP*/WT or the *gbss1* mutant (Figure 2). These data support the hypothesis that the effects of *gbss1* and overexpression of *OLE1* are synergistic with respect to leaf TAG accumulation.

Stacking of lipogenic factors WRINKLED1, DGAT1, and Cys-OLE1 into OG significantly increased leaf TAG accumulation.

**Figure 3:**
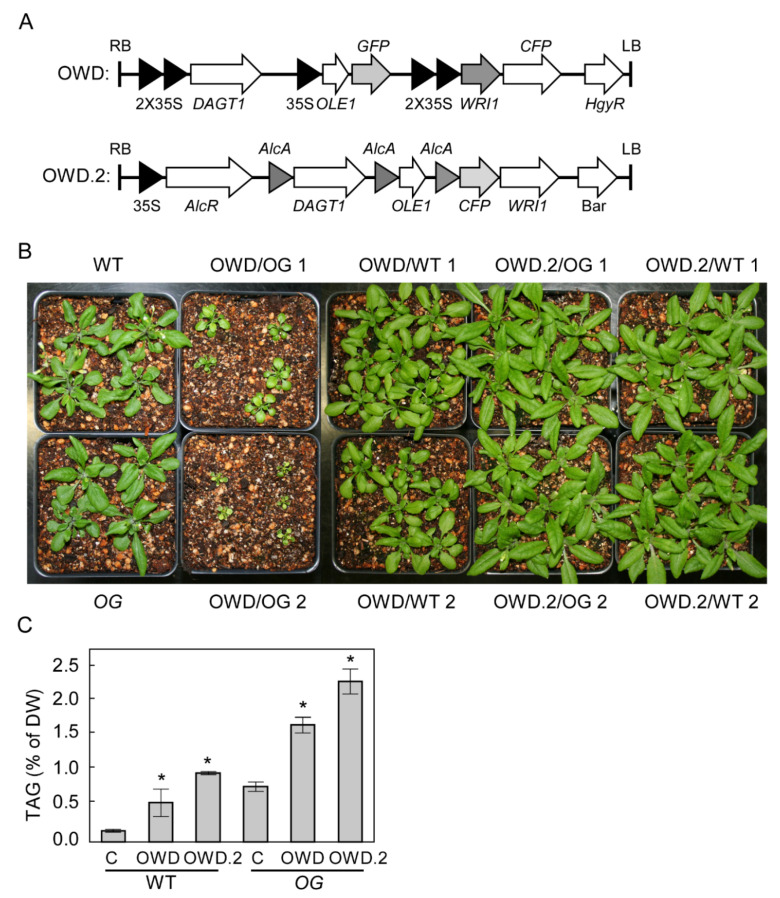
Stacking WRI1 and DGAT1 and Cys-OLE1 in *GO* significantly boosts its leaves oil content. (**A**) Schematic illustration of T-DNA construct designed for constitutive or ethanol inducible expression of *Cys-OLE*, *WRI1,* and *DGAT1* (OWD and OWD.2). (**B**) Representative phenotype of constitutive or inducible OWD transgenic plants. Two representative 8-week-old soil grown transgenic lines for constitutive (OWD) or inducible expression (OWD.2) of OWD in WT or *GO* background were shown. (**C**) Leaves TAG quantification for varied genotypes in (**B**). C is a non-transgenic plant. Values are means ± SE (*n* = 6). For the OWD.2 plant, TAG was measured after 5 days of induction by irrigating with 2% of ethanol solution. Asterisks (*) denote statistically significant differences compared with C (Student’s *t* test, *p* < 0.01).

Vegetative oil accumulation often has a negative impact on plant growth. However, *OG* grows indistinguishably from WT (Figure 3), which makes it suitable for further genetic manipulation to hyper-accumulate TAG in leaves.

To increase leaf TAG accumulation, three genes encoding three lipogenic factors, Cys- *OLE1*, *WRI1*, and *DGAT1*, were assembled into a single T-DNA (Figure 3A). Based on transient expression assays in *N. benthamiana*, in which WRI1 was found to be more potent as an N-terminal fusion with cyan fluorescent protein (CFP) (Supplementary Figure S3), *CFP*-*WRI* was incorporated into the T-DNA construct. To monitor the levels of Cys-OLE1, GFP was fused in frame at its C terminus. The resulting *Cys*-*OLE1*-*GFP*, *CFP*-*WRI1,* and *DGAT1* construct, referred as OWD hereafter, (Figure 3A) was validated by transient expression in *N. benthamiana* leaves. Both WRI1 and Cys-OLE1 exhibited strong expression 2 days after infiltration as monitored by fluorescence signal localization in the nucleus and oil bodies, respectively (Supplementary Figure S4A). At 4 days after infiltration, TAG accumulation of OWD-infiltrated leaves reached 3.3% (DW), which corresponds to a 60-fold increase compared with empty vector controls (Supplementary Figure S4B). After validation, OWD was transformed into the Arabidopsis WT (WT) and *OG* backgrounds. For each transformation, 10 independent transformants were screened for elevated TAG in the T2 generation, and 3 homozygous transgenic lines with highest TAG levels were advanced to the T3 generation. Ectopic overexpression of OWD was detrimental to growth, but more so in the *OG* background than in WT (Figure 3B). We also observed that OWD transgenic plants showed some reduced growth, delayed flowering, and reduced fecundity (data not shown). TAG accumulation in leaves of *OG* OWD transgenic plants were 1.64% on dry weight basis vs. 0.6% in WT leaves of OWD transgenics (Figure 3C).

To further explore the feasibility of this strategy in boosting vegetative TAG, we created a construct in which WRI1 was placed under the control of ethanol-inducible promoter, and OLE1 and DGAT1 were under strong constitutive control. As before, the ethanol-inducible TAG construct was first validated by transient expression in *N. benthamiana*. One day after agroinfiltration, the expression of *WRI1* was induced by drenching roots with 1% or 2% of ethanol. Four days of ethanol induction elevated TAG levels 15-fold, whereas without ethanol induction, *AlcA*:*WRI1*-infiltrated leaves barely accumulated any TAG. Interestingly, incubation of roots in 2% ethanol resulted in similar leaf TAG accumulation to that resulting from 4 days of agroinfiltration with 35S:*WRI1* (Supplementary Figure S5). We next constructed OWD.2, in which each of the three genes were placed under the control of the *AlcA* promoter (Figure 3A) and transformed it into the Arabidopsis WT and *OG* backgrounds, respectively. As expected, both WT and *OG* OWD.2 transgenic plants did not show any abnormalities during development in the absence of ethanol (Figure 3B). TAG accumulation in the leaves of 8-week-old WT and *OG* OWD.2 transgenic plants reached 1% and 2.26%, respectively, after 5 days induction with 2% of ethanol solution vs. 0.6% and 1.64% TAG in constitutive expression of OWD in WT and *OG* (Figure 3C), providing support for inducible expression as a viable strategy for increasing vegetative TAG accumulation.

## 3. Discussion

Here, we show that an *Arabidopsis* WT *OLE1*-GFP transgenic line (*OG*) [22] contains a single T-DNA insertion within a starch synthase gene, *GBSS1*, which results in a significant reduction of amylose content. The elevated leaf TAG phenotype characteristic of *OG* results from the serendipitous disruption of *GBSS1* by the *OLE1-GFP* T-DNA. This is supported by phenocopying the elevated leaf TAG of OG by combining the overexpressing *OLE1-GFP* in a *gbss1* background (GABI_914G01), which is higher than either *OLE1-GFP*/WT or the *gbss1* mutant alone.

As the major non-structural carbohydrate in plants, starch serves as an important transient form of carbon storage that fuels plant metabolism and growth in the absence of photosynthesis. Starch is almost entirely made up of amylose and amylopectin [25]. GBSS1 is the starch synthase that catalyzes synthesis of amylose in chloroplast starch granules. TAG synthesis and starch synthesis compete for carbon flux [26], so the overexpression of *OLE1-GFP* in a *gbss1* mutant background results in higher TAG accumulation most likely because of reduced competition for carbon. An alternative possibility is that monoacyl lipids (non-esterified fatty acid and lysophospholipids) that under normal conditions strongly associate with amylose could be channeled towards TAG synthesis in the *gbss1* mutant. However, TAG content in the *gbss1* mutant is no different from that of WT, indicating that the reduction of amylose alone is not sufficient to increase TAG accumulation.

Mutations such as *sdp1,* which is deficient in a triacylglycerol lipase, and *tgd1,* which is a leaky mutant for TRIGALACTOSYLDIACYLGLYCEROL1 (TGD1) in which approximately half of its seeds abort, resulting in high-leaf-TAG phenotypes, require sugar supplementation for germination on Murashige and Skoog (MS) medium [14,27]. However, *OG,* which also shows elevated vegetative TAG levels, grows from seed to seed indistinguishably from WT (Figure 3), making it a useful host for further increases in vegetative TAG accumulation. *Cys-OLE1*, *WRI1,* and *DGAT1* (OWD) were chosen for ectopic co-expression in *OG* because each of these genes have been shown to increase oil accumulation in vegetative tissues, and their co-expression has been shown to synergistically boost TAG accumulation [6,8,11,23,28,29,30]. In this study, transient co-expression of OWD in tobacco leaves increased TAG levels 60-fold relative to empty vector controls (Supplementary Figure S4), confirming our gene combination is effective in boosting oil in vegetative tissues.

Although constitutive co-expression of OWD in both Arabidopsis WT and *OG* plants further elevated TAG level to 0.6% and 1.64%, respectively, severely adverse effects on growth were observed (Figure 3). It was previously shown that *AGP*RNAi-*WRI1* overexpression delayed the development and expansion of cotyledons and leaves [11]. In the present study, ectopic overexpression of *OLE1-GFP* alone was also found to negatively affect silique development and corresponding seeds yields (Supplementary Figure S1). To circumvent these negative effects of constitutive overexpression of *OLE1* and *WRI1* on growth, ethanol-inducible expression of OWD was tested. Ethanol inducible gene expression in plants has many advantages when compared with other inducible systems, such as dexamethasone or heat shock, in minimizing negative impacts while maximizing induction sensitivity [31]. The OWD.2 T-DNA construct that placed all three genes under the control of the *AlcA* promoter was introduced into Arabidopsis WT and *OG* plants using floral dip transformation. As expected, both WT and *OG* OWD.2 transgenic plants established and grew similarly to WT transformants (Figure 3). Five days of ethanol induction further elevated TAG content to 1% and 2.26% in WT and *OG* OWD.2 transgenics, respectively, providing proof-of-concept for inducible production of oil in vegetative tissues.

## 4. Materials and Methods

### 4.1. Plant Materials and Growth Conditions

*Arabidopsis thaliana* wild-type (Col-0), *OG* transgenic line [22], and *gbss1* mutant (GABI_914G01 [24]) seeds were surface sterilized and selected on agar plates containing one-half-strength Murashige and Skoog salts. After 1 week, seedlings were transplanted to moist PM-15-13 AIS MIX (Lehle Seeds). All plants were grown in a 16 h-light/8 h-dark photoperiod (at a photosynthetic photon flux density of 250 μmol·m^−2^·s^−1^) at a 23°/19 °C light/dark temperature regime and 75% relative humidity.

Thermal asymmetric interlaced PCR (TAIL-PCR) was performed as described [32] with primers as listed in Supplemental Table S1 to identify the sequence flanking of the T-DNA in *OG*.

### 4.2. Genetic Constructs

*OLE1* (AT4G25140), *WRI1* (AT3G54320), and *DGAT1* (AT2G19450) were amplified by PCR from genomic DNA or seed complementary DNA of *Arabidopsis* by using the primer pairs listed in Table S1. The PCR products were cloned into the gateway pDONR/Zeo Vector (Invitrogen) by BP reaction and sub-cloned into the plant gateway binary vector: pGKPGWG [33], pGWB45, or pGWB414 [34] through LR reaction. Cys-*OLE1* attained as a custom synthesized sequence (GenScript, Piscataway, NJ, USA) was amplified and cloned into pCHF3 between *Kpn*I and *Xba*I. For *GBSS1* RNAi, 207bp of its coding region was cloned into vector pRNAi-GG [35]. For assembling OWD, *DAGT1* or *Cys-OLE1* expression cassette containing 35S promoter, gene, and terminator was amplified from the corresponding plant binary vector and sub-cloned into the *Pme*I and *HindIII* sites of pGWB45 containing *WRI1* through in-fusion (Clontech), respectively. For ethanol inducible expression of three genes (OWD.2), *GFP-WRI1* fragment of pMDC43 containing *WRI1* was excised using *Kpn*I and *Pac*I and inserted into the corresponding sites in pBJ36_AlcA; *DAGT1* and cys-*OLE1* were amplified by PCR, excised using *Kpn*I and *Xba*I, and inserted into pBJ36_AlcA, respectively. Then, *GFP-WRI1*, *DAGT1,* or Cys-*OLE1* expression cassette (each comprising *AlcA* promoter, gene, and terminator amplified from the corresponding pBJ36 vector) were one by one sub-cloned into *Not*I of pMBLART_AlcR by in-fusion.

RNA isolation and quantitative real time PCR *(qRT-PCR)* were carried out as previously described [36]. Gene-specific primers for *GBSS1* and *F-box* (At5g15710) were used as a reference gene (Table S1).

### 4.3. Preparation of Leaf Starch Granules and Iodine Staining

Leaf starch granules were isolated according to Zeeman et al. [37].

Transient gene expression in N. bethamiana was performed as described [38]. For TAG quantification, 8 leaves from 4 plants (2 from each) were agroinfiltrated with the same construct combination.

TAG quantification was performed as previously described [16].

## Data Availability

The data presented in this study are available in this article or supplemental materials here.

## Supplementary Materials

The following are available online at https://www.mdpi.com/2223-7747/10/3/513/s1, Figure S1: Characterization of WT *OLE1-GFP* transgenic (*OLE1-GFP*/WT) plants from independent transformation with the same genetic construct that was described to create the OG line. (A) OLE1-GFP/WT lines showed shorter siliques than OG. Arrows point to abnormal siliques of Ole1-GFP transgenic plants. (B) GFP fluorescence signal of *OLE1-GFP*/WT is significantly lower than OG. Bright light (above) and GFP fluorescence (below) of WT, *GO*, and a representative *OLE1-GFP*/WT line by fluorescence image analyzer (ImageQuant LAS4000); Figure S2: Oil accumulation in *N*. *benthamiana* leaves transiently expressing *OLE1-GFP*. EV is empty vector, and 1, 2, and 3 stand for three independent transient assay experiments. Values are means ± SE of measurements on 8 leaves from 4–5-week-old *N. benthamiana* plants infiltrated with *Agrobacterium* for 4 days. Asterisks denote statistically significant differences compared with EV (Student’s *t* test, *p* < 0.01); Figure S3: TAG levels in *N. benthamiana* leaves that were transiently transformed with EV (empty vector), *WRI1*, *CFP-WRI1* (CFP fused to the N terminus of WRI1), or *WRI1-CFP* (CFP fused to C terminus of WRI1). Values in this figure are means ± SE of measurements on 8 leaves from 4–5-week-old *N. Benthamiana* plants infiltrated with *agrobacterium* for 4 days. Asterisks denote statistically significant differences compared with *WRI1* (Student’s *t* test, *p* < 0.01); Figure S4: Laser scanning confocal images showed the co-expression of WRI1 and OLE1 in *N. benthamiana* epidermis cells transiently transformed with OWD (with *Cys-OLE1-GFP*, *CFP-WRI1*, and *DGAT1* contained in one engineered T-DNA). Bar = 50 µm. Values in this figure are means ± SE of measurements on 8 leaves from 4’5-week-old *N. Benthamiana* plants infiltrated with *agrobacterium* for 4 days; Figure S5: TAG levels in in *N. benthamiana* leaves that were transiently transformed with *AlcA:WRI1* (ethanol inducible) or 35S:*WRI1* (constitutive). Expression of *WRI1* was induced by irrigating with 0%, 1%, or 2% of ethanol solution for 4 days. Asterisks denote statistically significant differences compared with 0% of ethanol induction (Student’s *t* test, *p* < 0.01). Values in this figure are means ± SE (*n* = 8); Table S1: Primer sequences used in this study.
