# Supplementary material for: Ectopic Expression of OLEOSIN 1 and Inactivation of GBSS1 Have a Synergistic Effect on Oil Accumulation in Plant Leaves
Source: Plants (Basel). 2021 Mar 9;10(3):513. doi: 10.3390/plants10030513 (PMC8000217; doi:10.3390/plants10030513)
Supplement: Supplementary file 1 [file plants-10-00513-s001.pdf]

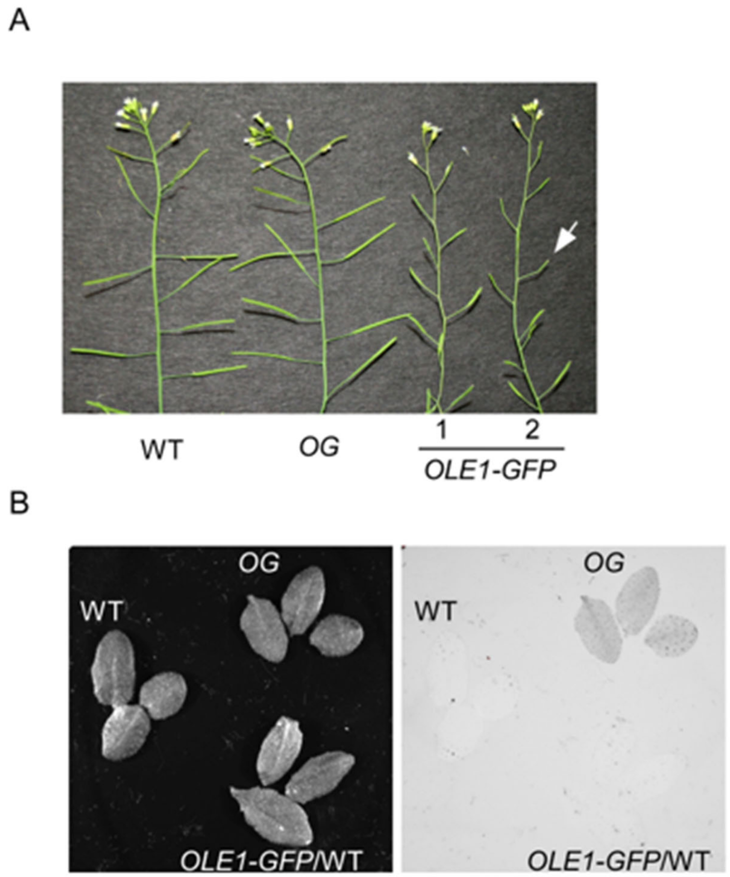

Supplemental Figure 1. Characterization of WT *OLE1-GFP* transgenic (*OLE1-GFP*/WT) plants from independent transformatio：n with the same genetic construct that was described to create the OG line. (A) *OLE1-GFP*/WT lines showed shorter siliques than OG. Arrows point to abnormal siliques of Ole1-GFP transgenic plants. (B) GFP fluorescence signal of *OLE1-GFP*/WT is significantly lower than OG. Bright light (above) and GFP fluorescence (below) of WT, *GO*, and a representative *OLE1-GFP*/WT line by fluorescence image analyzer (ImageQuant LAS4000).

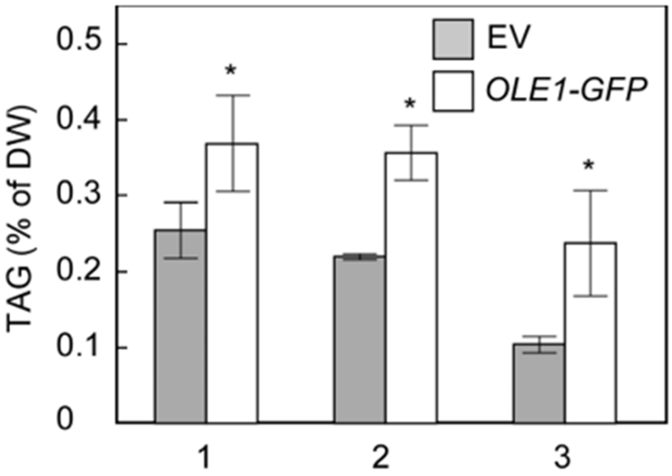

Supplemental Figure 2. Oil accumulation in *N. benthamiana* leaves transiently expressing *OLE1-GFP*. EV is empty vector, and 1, 2, and 3 stand for three independent transient assay experiments. Values are means ± SE of measurements on 8 leaves from 4 5-week-old *N. benthamiana* plants infiltrated with *Agrobacterium* for 4 days. Asterisks denote statistically significant differences compared with EV (Student's *t* test, $p < 0.01$).

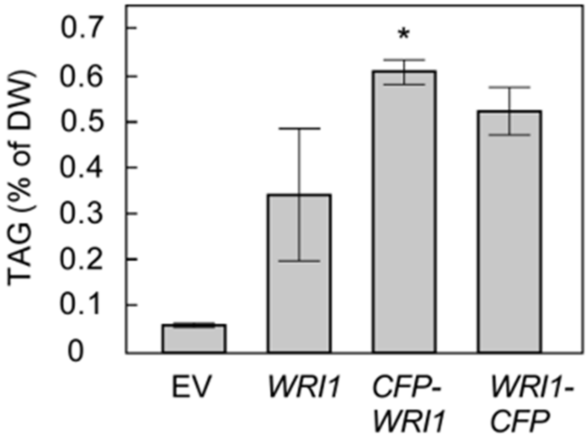

Supplemental Figure S3 TAG levels in *N. benthamiana* leaves that were transiently transformed with EV (empty vector), *WRI1*, *CFP-WRI1* (CFP fused to the N terminus of WRI1), or *WRI1-CFP* (CFP fused to C terminus of WRI1). Values in this figure are means ± SE of measurements on 8 leaves from 4 5-week-old *N. Benthamiana* plants infiltrated with *agrobacterium* for 4 days. Asterisks denote statistically significant differences compared with *WRI1* (Student's *t* test, $p < 0.01$)

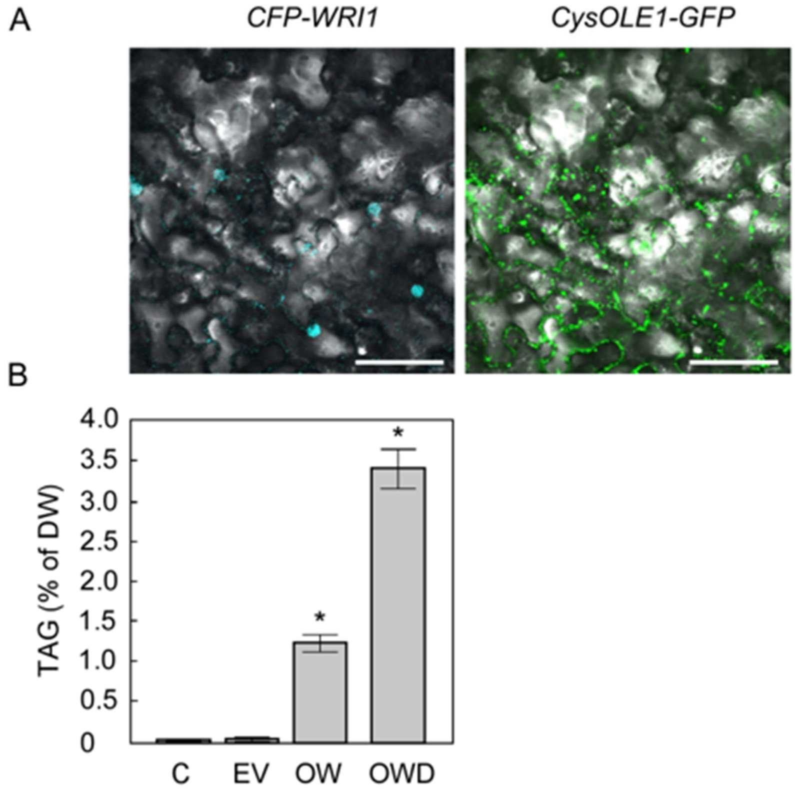

Supplemental Figure S4. Laser scanning confocal images showed the co-expression of WRI1 and OLE1 in *N. benthamiana* epidermis cells transiently transformed with OWD (with *Cys-OLE1-GFP*, *CFP-WRI1,* and *DGAT1* contained in one engineered T-DNA). Bar = 50 µm. Values in this figure are means ± SE of measurements on 8 leaves from 4 5-week-old *N. Benthamiana* plants infiltrated with *agrobacterium* for 4 days.

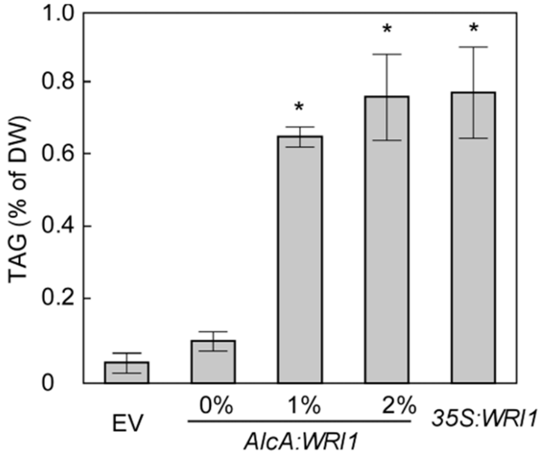

Supplemental Figure 5. TAG levels in in *N. benthamiana* leaves that were transiently transformed with *AlcA:WRI1* (ethanol inducible) or 35S:*WRI1* (constitutive). Expression of *WRI1* was induced by irrigating with 0%, 1%, or 2% of ethanol solution for 4 days. Asterisks denote statistically significant differences compared with 0% of ethanol induction (Student's *t* test, $p < 0.01$). Values in this figure are means ± SE ($n = 8$).

Supplemental Table 1. Primer sequences used in this study.

Gene	Primer pair sequences	Purpose
OLE1	GGGGACAAGTTTGTACAAAAAAGCAGG CTTCATGGCGGATACAGCTAGAGG and GGGGACCACTTTGTACAAGAAAGCTGG GTCAGTAGTGTGCTGGCCACCA	pGKPGWG
WRI1	GGGGACAAGTTTGTACAAAAAAGCAGG CTTCATGAAGAAGCGCTTAACCACTTC and GGGGACCACTTTGTACAAGAAAGCTGG GTCTTATTCAGAACCAACGAACAAGCC	pGWB45
WRI1	GGGGACAAGTTTGTACAAAAAAGCAGG CTTCATGAAGAAGCGCTTAACCACTTC and GGGGACCACTTTGTACAAGAAAGCTGG GTCGGACCAAATAGTTACAAGAAACCG AGG	pMDC85
DGAT1	GGGGACAAGTTTGTACAAAAAAGCAGG CTTCATGGCGATTTTGGATTCTGCTG and GGGGACCACTTTGTACAAGAAAGCTGG GTCTCATGACATCGATCCTTTTCGG	pGWB414
Cys-OLE expression module	GGCCAGTGCCAAGCTTGTGGAGCACGAC ACACTTGTCT and GCAGGCATGCAAGCTTTGCCAAGCTAGC TTGATGCATG	pGWB45
DAGA1 expression module	AAACACTGATAGTTTAAACGCAGGTCCC CAGATTAGCCTTTTC and TCCCGCCTTCAGTTTAAACAGTTAGCTC ACTCATTAGGCACCC	pGWB45
Cys-OLE1	CTTGGGTACCATGGCGTGTCATTATGGT CAACAAC and GCTATCTAGA GCTGGTCTGGCTACCTGC	pCHF3 and pBJ36_AlcA
GBSS1	ACCAGGTCTCAGGAGTCAGGCTGCTGTT GGACTTCC and ACCAGGTCTCATCGT GCCTTCCCTGGGAACTTCTCTT	pRNAi-GG
DGAT1	CTTGGGTACCATGGCGATTTTGGATTCT GCTG and GCTATCTAGATGACATCGATCCTTTTCG GTTCATC	pBJ36_AlcA
WRI1 ethanol inducible expression module	GACCTGCAGGCGGCCGCATATGCGGGAT AGT and ATCACTAGTGCGGCCTCCTGCTGAGCCT CGACATGTTGTCGC	pMBLART_AlcR
Cys-OLE1 ethanol inducible expression module	GACCTGCAGGCGGCCGCATATGCGGGAT AGT and CCCGCATATGCGGCCTCCTGCTGAGCCT CGACATGT	pMBLART_AlcR
DGAT1 Ethanol inducible expression module	GACCTGCAGGCGGCCGCATATGCGGGAT AGT and CCCGCATATGCGGCCTCCTGCTGAGCCT CGACATGT	pMBLART_AlcR
GBSS1	CATGGACCAAGACTTCTCCTG and TCACTTCCCGCCACATTAAG	qRT-PCR
F-box	TTTCGGCTGAGAGGTTCGAGT and GATTCCAAGACGTAAAGCAGATCAA	qRT-PCR
LBa1	GGTTCGGACTCTAGCTAGAGTCAAG	TAIL-PCR
LBb1	GATTGAATCCTGTTGCCGGTCTTG	
LSA1	GTAATACGACTCACTTAGGGCACGCGTG GTCGACGGCCCGGGCTGC	
SSAHind	AGCTGCAGCCCGGGCC	
SSAEco	AATTGCAGCCCGGGCC	
AP1	GTAATACGACTCACTATAGGGC	
AP2	TGGTCGACGGCCCGGGCTGC	

LAD1-1	ACGATGGACTCCAGAGCGGCCGC
AC1	ACGATGGACTCCAGAG
RB-0b	CTTGACGAGTTCTTCTGAGCGGGACTC
RB-1b	ACGATGGACTCCAGTCCGGCCGAGTCAA<br>GCAGATCGTTCAAACATTTGGC
RB-2b	ATCCTGTTGCCGGTCTTGCGATG
